# Effectiveness of suprascapular nerve block for the treatment of frozen shoulder: A protocol of systematic review

**DOI:** 10.1097/MD.0000000000020987

**Published:** 2020-07-02

**Authors:** Shou-feng Wang, Tian-shu Wang, Jian-an Li, Zhao-chen Tang, Xiao-feng Qiao

**Affiliations:** a First Ward of Orthopedics Department; b Second Ward of Orthopedics Department, First Affiliated Hospital of Jiamusi University; c School of Clinical Medicine, Jiamusi University, Jiamusi, China.

**Keywords:** effectiveness, frozen shoulder, safety, suprascapular nerve block

## Abstract

**Background:**

This study will appraise the effectiveness and safety of suprascapular nerve block (SNB) for the treatment of frozen shoulder (FS).

**Methods:**

This study will incorporate studies relevant to SNB on FS. Articles will be searched in the electronic databases (MEDLINE, EMBASE, CINAHL, Web of Science, PsycINFO, Cochrane Library, WANGFANG, and CNKI) from inception to the present. In addition, this study will also retrieve conference proceedings and reference lists of included studies. All literature source searches will not be restricted by date and language. The Cochrane Risk of Bias Tool will be utilized to evaluate the quality of retrieved trials. Data will be collected independently by 2 authors. All collected data will be analyzed by RevMan 5.3 software.

**Results:**

This study will synthesize the most recent published high quality trials on assessing the effectiveness and safety of SNB in treating FS.

**Conclusion:**

The findings of this study will provide a genuine understanding and helpful evidence to determine whether SNB is effective or not in treating FS.

**Study registration number:**

INPLASY202050084.

## 1 Introduction

Frozen shoulder (FS), also known as adhesive capsulitis, is a very common progressive shoulder disorder,^[1–3]^ which often causes shoulder pain and functional disability.^[4–6]^ It is estimated that such condition affects approximately 2% to 4% of the general population.^[7]^ Despite the increasing understanding of its underlying pathology, it is still poorly understood,^[8]^ and no optimal treatment strategy for FS is recommended.^[9–11]^

Suprascapular nerve block (SNB) is reported to treat FS effectively.^[12–26]^ However, all conclusions drawn are based on the individual study, and there are still inconsistent conclusions regarding this issue.^[12–26]^ In addition, no systematic review performed this topic. Thus, this study will systematically and comprehensively assess the effectiveness and safety of SNB in treating FS.

## 2 Methods and analysis

### 2.1 Study registration

We have registered this protocol on INPLASY202050084. We report this study according to the Preferred Reporting Items for Systematic Reviews and Meta-Analysis Protocol statement.^[27]^

### 2.2 Study eligibility criteria

Patients who were diagnosed as FS will be included, in spite of educational background, sex, race, and severity of FS.

This study will include randomized controlled trials (RCTs) investigating the effectiveness and safety of SNB in treating FS. We will exclude non-clinical trials, uncontrolled studies, and non-RCTs.

We will include patients who receive SNB as interventional management. Any interventions can be utilized as comparators, but not SNB.

The primary outcome is shoulder pain intensity, as reported by primary trial, such as visual analog scale. The secondary outcomes include functional ability (as measured by Oxford Shoulder Score or other relevant scales), shoulder range of motion (as reported by Passive Range of Motion, or other related scales), health related quality of life (as evaluated by 3 level EuroQol five-dimensional questionnaire or other associated tools), and adverse events.

### 2.3 Search strategy

An experienced librarian with expertise in systematic reviews has been consulted to develop the search strategy from 2 search methods. All literature searches will not be limited by publication date and language. A primary search will be performed in the electronic databases (MEDLINE, EMBASE, CINAHL, Web of Science, PsycINFO, Cochrane Library, WANGFANG, and CNKI) from inception to the present. A detailed search strategy of MEDLINE is built in Table 1, and similar search strategies are adapted to the other electronic databases.

**Table 1:**
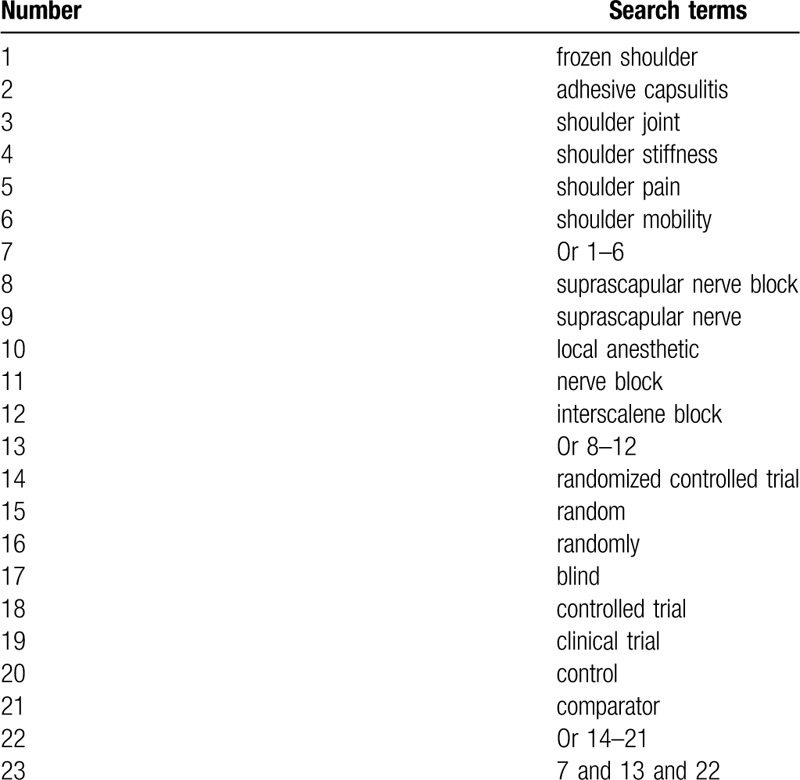
Search strategy of MEDLINE.

A secondary search will be performed in conference proceedings, ongoing trials from clinical trial registry, and reference lists of relevant reviews.

### 2.4 Data collection and analysis

#### 2.4.1 Selection of studies

All searched citations will be imported into EndNote X7 and duplicates will be eliminated. Two authors will independently and thoroughly investigate: titles and abstracts; and full texts of potential trials in 2 separate stages. The whole process will abide to all eligibility criteria, and will be presented in a flow diagram. Any confusion will be clarified by a third author through discussion.

#### 2.4.2 Data extraction and management

Two authors will carry out data extraction based on the pre-designed standardized data extraction form independently and separately. Any dissimilarity will be disentangled by a third author through discussion or consultation. We will extract the following information: study information (e.g., title, primary author, year of publication), patient demographics (e.g., race, age, and eligibility criteria), trial setting, trial design, trial methodological quality, details of treatments and controls, primary and secondary outcomes, safety, and other important information.

#### 2.4.3 Missing data dealing with

All missing data or insufficient data will be requested by contacting primary study authors through email. If it is not available, we will analyze extracted data only.

### 2.5 Study methodological quality assessment

All study methodological quality of eligible quantitative research trials will be appraised using Cochrane risk of bias tool. This tool includes 7 domains, and we will rate each one as low, unclear or high risk of bias. Any disagreements will be solved by a third author through consultation.

### 2.6 Statistical analysis

We will undertake statistical analysis using RevMan 5.3 software (Cochrane Community, London, UK). All continuous outcome values will be estimated as weighted mean difference (MD) or standard MD and 95% confidence intervals (CIs), and all dichotomous outcome values will be expressed as risk ratio and 95% CIs. We will examine statistical heterogeneity across trials using *I*^2^ test. *I*^2^ ≤ 50% exerts minor heterogeneity, and a fixed-effects model will be employed; *I*^2^ > 50% reveals significant heterogeneity, and a random-effects model will be placed. We will conduct a meta-analysis if minor heterogeneity is examined across sufficient data on the same outcome indicator. On the other hand, if substantial heterogeneity is detected, we will perform subgroup analysis and meta-regression to explore heterogeneity sources.

### 2.7 Additional analysis

We will perform a subgroup analysis based on the different study information, patient characteristics, study methodological quality, and details of treatment and control.

We will carry out a sensitivity analysis to examine the stability of study results by eliminating low quality trials.

This study will investigate reporting bias by funnel plot if over 10 eligible trials are included.^[28,29]^

### 2.8 Ethics and dissemination

Since no individual patient data will be extracted from this study, thus it does not require ethical approval. We will publish this study on a peer-reviewed journal.

## 3 Discussion

Although previous studies have reported the efficacy and safety of SNB for the treatment of FS, no systematic review has addressed this topic. Thus, to our best knowledge, this study will be the first to synthesize the available evidence on SNB in treating FS. The findings of this study may provide evidence to clinicians; inform policy-makers in developing appropriate guidelines for patients with FS; and guide future research concerned this issue.

## Author contributions

**Conceptualization:** Shou-feng Wang, Jian-an Li, Zhao-chen Tang, Xiao-feng Qiao.

**Data curation:** Shou-feng Wang, Tian-shu Wang, Xiao-feng Qiao.

**Formal analysis:** Shou-feng Wang, Tian-shu Wang, Jian-an Li, Zhao-chen Tang.

**Funding acquisition:** Xiao-feng Qiao.

**Investigation:** Xiao-feng Qiao.

**Methodology:** Shou-feng Wang, Tian-shu Wang, Jian-an Li, Zhao-chen Tang.

**Project administration:** Xiao-feng Qiao.

**Resources:** Shou-feng Wang, Tian-shu Wang, Jian-an Li, Zhao-chen Tang.

**Software:** Shou-feng Wang, Tian-shu Wang, Jian-an Li, Zhao-chen Tang.

**Supervision:** Xiao-feng Qiao.

**Validation:** Shou-feng Wang, Tian-shu Wang, Jian-an Li, Zhao-chen Tang, Xiao-feng Qiao.

**Visualization:** Shou-feng Wang, Tian-shu Wang, Xiao-feng Qiao.

**Writing – original draft:** Shou-feng Wang, Tian-shu Wang, Jian-an Li, Zhao-chen Tang, Xiao-feng Qiao.

**Writing – review & editing:** Shou-feng Wang, Tian-shu Wang, Zhao-chen Tang, Xiao-feng Qiao.
